# The Seroprevalence, Risk Factors, and Clinical Profile of Hepatitis D in Omani Patients with Chronic Hepatitis B: A Multicenter Cross-Sectional Study

**DOI:** 10.3390/jcm14197089

**Published:** 2025-10-08

**Authors:** Khalid M. AlNaamani, Wafa Al-Tamtami, Mohamed El-Kassas, Heba Omar, Abdullah AlKalbani, Bola. R. Kamath, Halima Alshuaili, Amal Anwar, Alya AlKalbani, Hajer AlShukaili, Malak AlSawafi, Muneera AlShukaili, Siham AlSinani

**Affiliations:** 1 Department of Medicine, Division of Gastroenterology and Hepatology, The Medical City for Military and Security Services, Muscat 111, Oman; hebaomar1202@hotmail.com (H.O.); ahs92@hotmail.com (A.A.); brkamat@gmail.com (B.R.K.);; 2 Department of Medical Laboratory, Division of Medical Microbiology, The Medical City for Military and Security Services, Muscat 111, Omanjoory928@gmail.com (H.A.); 3 Endemic Medicine Department, Faculty of Medicine, Helwan University, Cairo 11795, Egypt; 4 Applied Science Research Center, Applied Science Private University, Amman 11931, Jordan; 5 Endemic Medicine and Hepatology Department, Faculty of Medicine, Cairo University, Cairo 11562, Egypt; 6 Department of Nursing, The Medical City for Military and Security Services, Muscat 111, Oman; 7 Department of Child Health, Sultan Qaboos University Hospital, University Medical City, Muscat 123, Oman; sihamsinani@gmail.com; 8 Department of Child Health, Sultan Qaboos University Hospital, Sultan Qaboos University, Muscat 123, Oman

**Keywords:** hepatitis B virus, hepatitis D virus, seroprevalence, risk factors, Oman, fibrosis

## Abstract

**Background**: Since the introduction of the hepatitis B virus (HBV) vaccination program in Oman in 1990, the HBV prevalence has markedly decreased. However, hepatitis D virus (HDV) infection, which is associated with progressive liver disease in patients with chronic HBV, remains understudied in the Omani population. This study aimed to estimate HDV’s seroprevalence, characterize its virological and clinical features, and identify factors associated with anti-HDV positivity among adult Omani patients with chronic HBV infection. **Methods**: We conducted a multicenter cross-sectional study in 2024 at two referral hospitals and two polyclinics in Oman. Adult Omani patients with chronic HBV (HBsAg-positive for >6 months) were enrolled. Demographic, clinical, laboratory, imaging, and elastography data were collected. The total anti-HDV antibodies were tested using an ELISA; HDV RNA was tested for anti-HDV-positive or equivocal results. Liver Fibrosis was assessed non-invasively through liver stiffness measurement (LSM) using vibration-controlled transient elastography (VCTE); FibroScan^®^ and clinical evaluation. Ridge (penalized) logistic regression identified predictors independently associated with anti-HDV positivity. **Results**: Among 639 patients (59.3% male; mean age of 46.6 ± 8.8 years), 36 patients were anti-HDV-positive, resulting in an HDV seroprevalence of 5.6% (95% CI: Exact 3.98–7.71; Wilson 4.10–7.70). Only one anti-HDV-positive patient had detectable HDV RNA, which became undetectable on follow-up without HDV treatment. The anti-HDV-positive patients were more frequently female and had a higher frequency of prior blood transfusions. In a penalized multivariable analysis, blood transfusions were independently associated with anti-HDV positivity (OR of 19.94), whereas male sex was associated with lower odds of being anti-HDV-positive (OR of 0.15). All the anti-HDV-positive patients had mild fibrosis (F0–F1). **Conclusions**: Our study demonstrated an anti-HDV prevalence of 5.63% among adult Omani patients with chronic HBV infection, while active viremia appeared to be rare. Blood transfusions were the main identified risk factor. Given the very low HDV viremia, targeted screening of higher-risk groups may be efficient.

## 1. Introduction

Hepatitis delta virus (HDV) is a defective hepatotropic virus with a small, single-stranded ribonucleic acid (RNA) genome [1]. Lacking its own envelope proteins, HDV depends on the hepatitis B virus (HBV): it borrows the three HBV surface proteins (HBsAg) to acquire an envelope, exit hepatocytes, and enter new hepatocytes. Therefore, productive infection and person-to-person transmission require the presence of HBsAg. In contrast, once in the hepatocyte, HDV replicates independently of HBV, using host RNA polymerase II. HDV RNA and delta antigen can persist through cell division even without HBV [2].

Co-infection of HBV and HDV can occur at the same time or as an HDV superinfection of a patient with HBV. Simultaneous infection with HBV and HDV is associated with the clearance of both viruses in most cases, whereas HDV superinfection in patients with HBV tends to persist, leading to a chronic infection and accelerated progression to cirrhosis, hepatic decompensation, and hepatocellular carcinoma (HCC), more rapidly than in HBV or HCV monoinfection [3,4,5].

The prevalence of chronic HDV infection, as estimated by the World Health Organization (WHO), is around 4.5% of patients with an HBsAg infection [6]. This prevalence has marked geographical variations, with the highest prevalence in Mongolia, China, Pakistan, Iran, Turkey, Romania, and sub-Saharan Africa [7]. A recent study by the Polaris Observatory Collaborators evaluating HBsAg-positive patients in 25 countries demonstrated the highest anti-HDV prevalence of 60% in Mongolia. However, after adjustment for the total number of patients with HBV and positive HDV RNA, China had the highest absolute number of HDV RNA-positive cases [8].

Since HDV is dependent on HBV for cell entry and infectivity, HBV vaccination is expected to decrease the risk of HDV infection. In a multicenter Italian study, Gaeta et al. demonstrated a significant decline in HDV prevalence, reporting a rate of 8.3%, compared with 23% and 14% documented in two prior multicenter surveys conducted in 1987 and 1992, respectively [9]. With the adoption of HBV vaccination in many countries around the globe, the incidence of new cases of HDV, as well as its prevalence, is expected to decrease markedly.

In Oman, the estimated chronic HBV prevalence was around 2–7% before a nationwide vaccination program was introduced in 1990 [10]. Subsequent studies revealed a marked reduction in the HBV prevalence [10,11,12], with blood donor positivity declining from 4% in 1990 to 1% in 2010 [11]. However, despite Oman’s proximity to HDV-endemic countries, local data on the HDV prevalence remain scarce. The aim of this study is to determine the prevalence of HDV in adult Omani patients and to characterize the clinical features of patients with an HBV/HDV co-infection. It is predicted that prevalence data will provide important information for the selection of an appropriate HDV screening strategy in Oman.

## 2. Methods

### 2.1. Study Design and Patient Population

This multicenter cross-sectional study was conducted from January through December 2024 at two referral hospitals within the Medical City for Military and Security Services (MCMSS) and two affiliated polyclinics serving the northern and interior regions. These two hospitals and two polyclinics accept patients from all over Oman. Adult Omani patients with a chronic HBV infection, defined as persistent HBsAg positivity for more than six months, irrespective of their HBVDNA and under routine follow-up at one of the participating sites were included.

We excluded patients with isolated anti-HBc and those with incomplete data required to determine the primary outcome.

### 2.2. Data Collection

Demographic, clinical, and risk factor information (including information on prior transfusions, surgeries, travel to regions with high HDV endemicity, participants’ family history, alcohol consumption, and smoking) were collected from the clinical record and confirmed at clinic visits where feasible. Laboratory investigations, including liver biochemistry tests and serologies for HBV, HCV, HDV, and human immunodeficiency virus (HIV), were conducted. Patients with a positive or equivocal total anti-HDV result were tested for HDV RNA as described below. Radiological data were also collected, including an ultrasound and/or computer tomography of the abdomen and liver stiffness measurement (LSM) using vibration-controlled transient elastography (VCTE; FibroScan^®^, Echosens, Paris, France) which was performed in all patients.

### 2.3. Serological Tests

An HBV infection was diagnosed based on persistent HBsAg positivity for more than six months, irrespective of serum HBV DNA levels. HBV DNA was assessed using the COBAS^®^ AmpliPrep/COBAS^®^ TaqMan^®^ HBV Test, v2.0 (Roche Diagnostics, Branchburg, NJ, USA), which has a lower limit of detection of 15 IU/mL and a linear dynamic range up to 1.7 × 10^8^ IU/mL.

An HDV infection was diagnosed when total anti-HDV antibodies, predominantly IgG, were detected using a competitive enzyme-linked immunosorbent assay (ELISA) based on a two-step methodology. Testing was performed using a commercially available kit (HDV Ab ELISA, Dia.Pro Diagnostic Bioprobes S.r.l., Milan, Italy). According to the manufacturer, the assay demonstrates sensitivity and specificity greater than 98%. The tests were performed at the Central Public Health Laboratory (CPHL), Oman, and the anti-HDV antibody results were interpreted based on the ratio of the sample optical density at 450 nm (OD450) to the assay cut-off value (Co/S ratio). A Co/S ratio of <0.9 was considered negative, 0.9–1.1 equivocal, and >1.1 positive for anti-HDV antibodies. In patients with borderline, equivocal, and positive serology, HDV RNA testing was performed to confirm an active infection.

Testing for HDV RNA was performed through a real-time polymerase chain reaction (PCR) using the CFX96™ Real-Time PCR Detection System (Bio-Rad Laboratories, Hercules, CA, USA), following the manufacturer’s instructions. The assay’s lower limit of detection was 20 IU/mL (1.30 log IU/mL), with a quantification range from 50 to 3,500,000 IU/mL. Amplification was performed using reagents from Eurobio Green PCR Master Mix (Eurobio Scientific, Les Ulis, France) according to the manufacturer’s instructions.

### 2.4. Statistical Analysis

**Figure 1:**
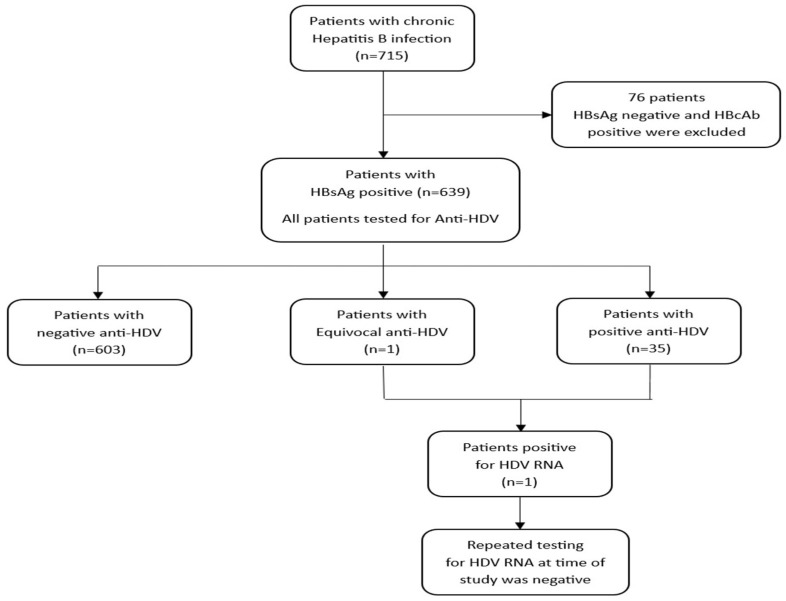
Flow diagram of HDV screening. Abbreviations: HBsAg, hepatitis B surface antigen; HBcAg, hepatitis B core antigen; anti-HDV, antibody to hepatitis D virus; HDV RNA, ribonucleic acid from hepatitis D virus.

The sample size was calculated based on the global prevalence of hepatitis D which is approximately 5%. Using a desired confidence level of 95% and precision of ±3%, the sample size needed is approximately 203 patients. For our study, a total of 715 patients with chronic hepatitis B who were on regular follow up at the participating sites were screened, of whom 76 were positive for anti-HBc and negative for HBsAg and were excluded. The study ultimately included 639 HBsAg-positive adults from all the participating centers (Figure 1).

Continuous variables were expressed as the mean ± the standard deviation (SD) or median (interquartile range, IQR), depending on the data distribution. Categorical variables were summarized as counts and percentages. Comparisons between the anti-HDV-positive and -negative patients were performed using an independent samples *t*-test or Mann–Whitney *U* test for continuous variables and the chi-square test or Fisher’s exact test for categorical variables, as appropriate.

The Wilson and Clopper-Pearson methods were used to estimate the 95% confidence intervals (CIs) of prevalence. These methods were selected because they provide more accurate CIs for proportions, particularly when data are sparse, such as in our small number of anti-HDV–positive cases.

To identify factors associated with anti-HDV positivity, multivariate model using ridge (L2) penalized logistic regression was performed. Ridge regression’s L2 penalty shrinks coefficients to prevent overfitting and enhance model stability, which is valuable when predictors are correlated, or sample size is small. To reduce the log-loss bias, 5-fold cross-validation was used to determine the regularization parameter (lambda). Candidate predictors were identified a priori by performing univariate logistic regression. Odds ratios (ORs) with 95% confidence intervals (CIs) where applicable were reported.

To assess the possibility of nonlinear age effects on anti-HDV positivity, we flexibly modeled age using natural cubic splines. The spline segment points (knots) were selected at the points of the 5th, 35th, 65th, and 95th percentile of the age distribution. To determine whether the correlation between age and anti-HDV positivity varies by gender due to physiological differences in immune response, we used the likelihood ratio test (LRT) to analyze the difference in the age effect on anti-HDV positivity. The LRT’s two models, with and without the interaction term (age * gender). Statistical significance was defined as a two-tailed *p*-value of <0.05.

All analyses were conducted using Python (version 3.13, Python Software Foundation).

### 2.5. Ethical Approval

Ethical approval for this study was obtained from the ethics committee of the Medical City for Military and Security Services (MCMSS), Muscat, Oman (ethical approval number AFMS-MREC: 001/2024). This ethical approval covered all the participating sites. The study was conducted in full compliance with the ethical principles outlined in the Declaration of Helsinki (2013 revision) issued by the World Medical Association. Information sheets were given to all the patients, and consent forms were obtained.

## 3. Results

### 3.1. Patient Characteristics

A total of 715 patients with chronic hepatitis B were screened, of whom 76 were positive for anti-HBc and negative for HBsAg were excluded. The study ultimately included 639 HBsAg-positive adults from all the participating centers (Figure 1). Of these, 379 patients (59.3%) were male, with a mean age of 46.6 ± 8.8 years. Thirty-six patients (5.6%) were anti-HDV-positive, one equivocal result and only one was HDV RNA-positive. This patient subsequently cleared the infection spontaneously without specific HDV treatment.

Five patients were co-infected with HCV and one patient with HIV. Seven patients with HBV had received a liver transplant, four of them for decompensated cirrhosis and three for HCC.

A total of 109 patients (17.1%) were receiving antiviral therapy for HBV. Tenofovir was the most commonly used medication, prescribed to 73 patients (67.0%), while 36 patients (33.0%) received entecavir. Among the anti-HDV positive patients, only three were on HBV treatment which was tenofovir. Significant fibrosis (≥F2) was identified in 27 patients (4.23%)**,** advanced fibrosis (≥F3) in 3 patients (0.47%), and cirrhosis (F4) in 13 (2.03%). Table 1 summarizes the demographic, clinical, and laboratory characteristics of the study population according to the participants’ anti-HDV status.

### 3.2. HDV Seroprevalence and Virological Findings

**Figure 2:**
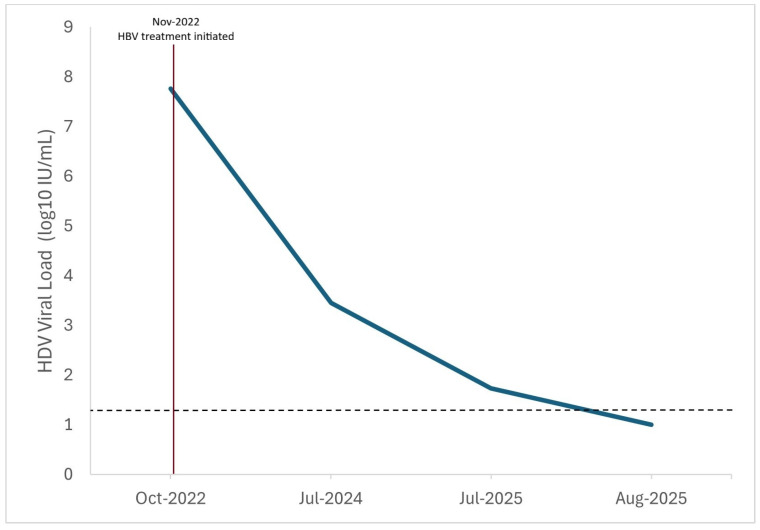
Decline in the HDV RNA level in a single patient with detectable HDV RNA. The dashed line (------) indicates the lower limit of detection (20 IU/mL = 1.3 Log_10_). The vertical line indicates the initiation of HBV treatment.

A total of 36 patients tested positive for total anti-HDV, corresponding to a prevalence of 5.6% (95% CI: Exact 3.98–7.71; Wilson 4.10–7.70). Of the 603 patients who tested negative, 582 (96.5%) had a Co/S ratio of <0.5, 20 (3.3%) were between 0.5 and 0.7, and 1 (0.2%) was between 0.7 and 0.9. Only one patient had a Co/S ratio in the equivocal range (0.9–1.1). The results of the Co/S ratio for the study population are presented in Figure 1. Only one patient had detectable HDV RNA, which subsequently became undetectable during a follow-up without HDV treatment (Figure 2). The majority of patients were HBeAg-negative (607 patients, 94.99%), and the mean HBV DNA level was 3.18 ± 1.42 log_10_ IU/mL.

A comparison between the anti-HDV-positive and -negative patients revealed that the anti-HDV-positive patients were relatively younger (mean age of 44.0 vs. 46.8 years, *p* = 0.07) and the majority were female (80.1%). A history of blood transfusion was significantly more frequent among anti-HDV-positive patients than anti-HDV-negative patients (8.3% vs. 0.5%). There were no significant differences in regard to complications such as the development of HCC, the requirement for liver transplantation, or HBV treatment (Table 1). Laboratory parameters, including the liver enzymes, bilirubin, albumin, and HBV DNA levels, were generally comparable between the anti-HDV-positive and -negative patients.

### 3.3. Fibrosis Assessment

Liver fibrosis was evaluated non-invasively through LSM using (VCTE; FibroScan^®^, Echosens, Paris, France). Predefined cut-off values were applied to categorize the fibrosis severity: significant fibrosis (F2 Metavir) was ≥7.2 kPa, advanced fibrosis (F3 Metavir) was ≥8.8 kPa, and cirrhosis (F4 Metavir) was ≥11 kPa [13].

Cirrhosis was defined based on either an LSM of more than 11 kPa or a clinician’s determination of the patient as cirrhotic in the medical records based on clinical status, liver chemistry tests, endoscopic and radiological findings. In cases where there was a discrepancy between the transient elastography score and the clinician’s assessment, the clinician’s evaluation was considered definitive.

Based on LSM and/or clinical evaluation, all the anti-HDV-positive patients had mild fibrosis (F0–F1) with a liver elasticity of <7.2 kPas, while significant or advanced fibrosis (≥F2) was only observed in anti-HDV-negative patients (Table 1). Of note, LSM is not validated for fibrosis assessment in post–liver transplant patients, particularly in those transplanted for HBV.

### 3.4. Factors Associated with Anti-HDV Positivity

The OR and 95% CIs for each independent variable in the univariate logistic regression model and OR for ridge regression are presented in Table 2. According to the model, history of blood transfusion is the most significant predictor of anti-HDV positivity (OR of 18.18, 95% CI: 3.53–93.56).

Male gender was associated with significantly lower odds of anti-HDV positivity (OR of 0.15, 95% CI: 0.06–0.35; *p* = > 0.001), indicating a protective effect not seen in females. For each additional year in age, the odds of being anti-HDV positive slightly decreased by 4% (OR of 0.96; 95% CI: 0.92–1.00), exhibiting a very modest protective effect. However, this association did not reach statistical significance, as the confidence interval included 1.0. History of travel to an endemic area did not have a statistically significant effect (OR 0.70, 95% CI: 0.36–1.37; *p* = 0.30), suggesting that it was not an independent risk factor in this patient population.

Age, gender, blood transfusion, and travel to HDV endemic areas were identified as candidate predictors for the ridge-penalized logistic regression model with history of blood transfusion being the strongest predictor with an OR of 19.94.

**Figure 3:**
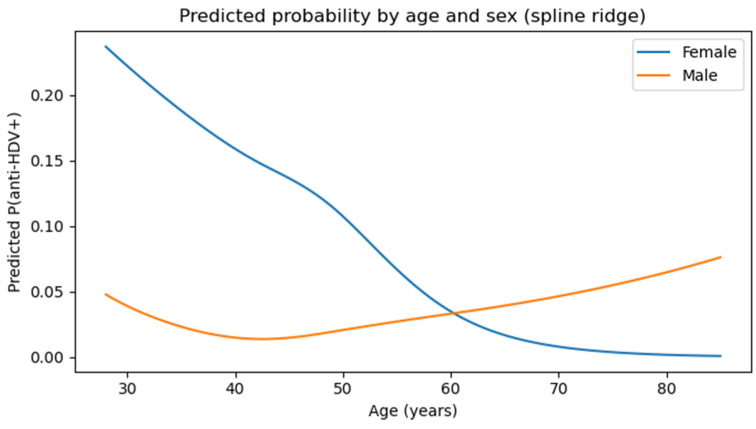
Predicted probabilities of anti-HDV positivity by age and gender.

The *p*-value for the LRT, which tested the effect of interaction between age and gender in predicting anti-HDV positivity, was 0.203, meaning there was no significant interaction. Nonlinearity was modeled using flexible age models including natural cubic splines, which did not multiply the parameters. The predicted probability of anti-HDV positivity with age and gender without a history of blood transfusion or travel to endemic areas is presented in Figure 3. There was no difference between males and females, suggesting that there is no significant interaction.

**Figure 4:**
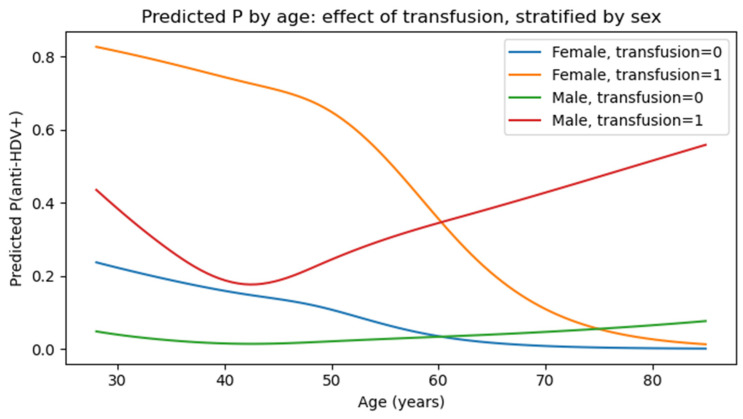
Predicted probabilities of anti-HDV positivity by age, stratified by gender and blood transfusion. Note: transfusion = 0 means no transfusion given, transfusion = 1 means transfusion given.

The likelihood of future anti-HDV positivity, considering both gender and blood transfusion status, is presented in Figure 4. The probability appears to be more strongly associated with the blood transfusion status, while there is no significant difference between the two genders across the age groups, which supports the lack of a significant interaction.

## 4. Discussion

Oman is geographically located near countries with a high HDV prevalence, such as Pakistan, Iran, and certain parts of India [6,14,15]. Over the past two decades, both medical tourism and leisure travel to and from these regions have increased, potentially contributing to HDV exposure in the Omani population, especially in individuals traveling for medical tourism [16]. This study included adult Omani patients from all regions of Oman and therefore provides a contemporary estimate of the HDV exposure among adult Omani HBsAg-positive patients, reporting an anti-HDV prevalence of 5.63%. This prevalence is lower than that reported in a study by Al-Dhahry et al. in 1994, which investigated the prevalence of HDV among high-risk patients such as those undergoing hemodialysis and renal transplants [17]. Al-Dhahry et al. identified anti-HDV in 7.7% of hemodialysis patients and 22.2% of renal transplant recipients, while none of the healthy HBsAg-positive controls tested positive for anti-HDV [17]. The previous study focused on high-risk patients, limiting its generalizability, whereas, the current study provides a broader and more representative sample of HBsAg-positive patients. The differences could also be explained by the use of the modern sensitive and specific assays to detect both HBV and HDV markers in the current study, as mentioned in the Methods Section, compared to the first-generation ELISA kits (Abbott Laboratories, Chicago, IL, USA) used in the previous study [18]. Improvements in infection control, the introduction of the HBV vaccination program in 1990 [11], and strict blood transfusion safety measures [19], have likely contributed further to this decline.

Compared to its neighboring countries, Oman’s HDV prevalence falls within an intermediate range. Recent regional data show an anti-HDV prevalence among HBsAg-positive patients ranging from 0% to 21.8%, with most estimates ranging between 2% and 10%, depending on the studied population [20]. Single-center studies reported HDV prevalences of 7.7% in Saudi Arabia, 6.6% in Iraq, and 0% in Yemen [20]. However, direct comparisons must be interpreted with caution due to differences in the sampling methods, population risk profiles, and assay characteristics [6].

Based on previous meta-analyses, the global prevalence of HDV infection is estimated at 4.5–13.0% among HBsAg-positive patients, and up to 14.6–18.6% in hepatology clinics, higher than observed in our study [6,21,22]. Our findings are of particular importance because the majority of our patients were recruited from hepatology clinics, where higher rates of detectable HDV RNA would typically be expected [6]. Several factors may explain our observations. Firstly, as mentioned previously, they may reflect a true reduction in active HDV infection in Oman, potentially due to improved HBV vaccination coverage, infection control measures, or reduced exposure to high-risk factors [11]. Secondly, the anti-HDV-positive patients may have previously cleared the virus, consistent with the known persistence of anti-HDV antibodies after HDVRNA clearance [2]. Thirdly, it is possible that differences in HDV genotypes across regions or over time may have contributed to the observed findings [23]. Unfortunately, HDV genotyping could not be performed in our patients due to the absence of detectable HDV RNA. Previous studies have identified eight different HDV genotypes that differ in their genomic sequence by at least 35% [24]. Compared with genotype 2, which is most commonly found in the Middle East, genotype 1 is associated with a higher risk of adverse outcomes and lower remission rates. This may partly explain the high rate of spontaneous HDV clearance and the absence of advanced fibrosis observed in our patients, assuming that our patients were predominantly infected with genotype 2 [22,25].

The presence of anti-HDV (IgG or total) antibodies in HBsAg-positive patients indicates prior exposure to HDV, while the presence of HDV RNA is required to confirm active replication [7]. A systematic review and meta-analysis by Stockdale et al. including over five thousand anti-HDV-positive patients reported detectable HDV RNA in 58.5% of cases (95% CI: 52.4–64.5), emphasizing that a significant number of anti-HDV-positive patients cleared the virus spontaneously [6].

The high prevalence of anti-HDV positivity but HDVRNA negativity underscores the importance of including both serologic and molecular testing when planning HDV surveillance programs. In a study by Salpini et al. evaluating the performance of various quantitative HDVRNA assays across 30 laboratories, several assays underestimated the viral load by more than 1 log_10_ IU/mL, and while the linearity was generally good, only a few assays maintained accuracy at low HDVRNA levels (<1000 IU/mL). Overall, the assay performance was heterogeneous, underscoring the importance of careful assay selection and interpretation of HDVRNA results in clinical practice [26].

We observed in our study that anti-HDV-positive patients were slightly younger than anti-HDV-negative patients. However, this age difference was only marginally non-significant after adjusting for other factors. The mean age of the entire study cohort was 46.61 ± 8.76, while the mean age of those with anti-HDV positivity was 44.00 ± 7.73, indicating that most of the patients were most likely infected with HBV before the introduction of HBV vaccination in Oman in 1990 which makes them susceptible to HDV infection [12]. The Italian experience described earlier demonstrated the important role of HBV vaccination in reducing the incidence and prevalence of HDV infection [9].

Our study is different from most previous studies, which demonstrated male gender to be a risk factor for HDV [27,28,29]. In our study, anti-HDV positivity was higher among female patients. The biological basis for gender differences in susceptibility to HDV infection remains unclear. This may be related to greater exposure to parenteral risk factors, such as blood transfusions or travel to endemic area, as indicated by our regression analysis [30]. Additional factor could be related to differential health-seeking behavior and higher utilization of healthcare services among women in Oman, where medical care is universally accessible and free of charge, potentially leading to increased detection of prior HDV exposure [31]. However, given the small number of anti-HDV-positive cases, these results must be interpreted cautiously and require further investigation in larger population-based studies.

Multiple previous studies highlighting that HDV can occur outside well-known high-risk groups [32,33]. A recent study from Spain by Palom et al. showed that 60% of those who tested positive for anti-HDV had no known risk factors for HDV infection [32].

Investigation of HDV risk factors in our study population revealed that a history of blood transfusion was a strong independent predictor of anti-HDV positivity (OR of 19.94), highlighting the key role of parenteral exposure in HDV transmission. Notably, this finding persisted despite Oman’s implementation of strict blood transfusion safety measures, including mandatory screening for HBV in all blood donors and adherence to international transfusion safety standards [34]. Collecting a detailed history of transfusions received before and after the introduction of systematic blood screening was not feasible in our cohort. The residual risk may be attributed to infections acquired before the introduction of strict screening programs or from exposure in private medical or emergency settings where strict testing may not have been consistently applied [35]. In addition, many patients from Oman had traveled to neighboring countries with high HDV endemicity for medical tourism. Although a history of travel to endemic areas was not statistically significant, the specific purpose of travel, whether for leisure or medical care, was not examined in detail. Similar trends in countries with strict transfusion safety standards indicate that transfusions received longer ago and lifetime exposure still contribute to HDV infection [28,36].

While co-infections with viruses such as HCV and HIV were not significantly associated with anti-HDV positivity in our study, this was most likely due to the low prevalence of HDV and the limited number of HBV co infected with HCV and HIV. Other studies have demonstrated a significantly higher prevalence, such as 15% among individuals with an HIV co-infection or a pooled OR of 10.0 among anti-HCV-positive patients [37,38]. This reinforces the importance of using context-specific screening strategies rather than depending on global risk assumptions, especially in regions where HDV’s epidemiology remains incompletely explored and inadequately characterized.

The results from laboratory investigations, including investigations of liver chemistry and HBV DNA levels, were similar between anti-HDV-positive and -negative patients. This was likely due to the absence of advanced liver fibrosis among anti-HDV-positive patients, as evaluated using Fibroscan. The liver elastography measurements showed that all the anti-HDV-positive patients had mild fibrosis (liver stiffness of <7 kPa), whereas significant fibrosis was observed exclusively in anti-HDV-negative patients. These findings were likely due to low HDV replication, a shorter active infection duration before spontaneous clearance of HDV RNA, and infection with the less virulent HDV genotype 2, which predominates in the Middle East. In contrast, persistent HDV RNA, particularly associated with genotypes 1 and 5, which are the most common in Europe and Africa, respectively, has been consistently identified as a major risk factor for progression to advanced fibrosis and increased mortality [39,40,41,42]. The low rate of detectable HDV RNA in our cohort further supports this observation.

Our study’s findings raise an important question: should we recommend screening for all HBsAg-positive patients or limit screening to high-risk patients? Screening all HBsAg-positive patients for HDV is recommended by the European Association for the Study of the Liver (EASL) and the guidelines of the Asian Pacific Association for the Study of the Liver (APASL) [4,43]. However, the low rate of active HDV replication observed in our cohort suggests that universal screening may not be cost-effective. In this context, a targeted screening strategy, such as the high-risk approach recommended by the AASLD, may be more appropriate [44]. This approach prioritizes testing among HBsAg-positive patients with known risk factors, including those with HIV co-infection, a history of intravenous drug use, or HBV patients with low or undetectable HBV DNA and persistently elevated ALT levels.

This study has some limitations, including the cross-sectional design, which prevented assessment of causality or changes over time in HDV infection. The presence of only a single patient with detectable HDV RNA restricted the evaluation of viral replication, the genotypes, and the disease severity. In addition, the unexpectedly low fibrosis stage (F0–F1) among anti-HDV–positive patients could not be fully explained or confirmed, and this uncertainty should also be considered a limitation. Additionally, the low prevalence of HIV and HCV co-infections and limited exposure data may have caused us to underestimate the associations with high-risk factors. Finally, despite using sensitive assays, variability in HDV RNA detection could have led to underestimation of the active infections.

Our study has several important implications. Firstly, it provides the first contemporary, nationally representative estimate of HDV exposure among HBsAg-positive patients in Oman, complementing the results from an older study of high-risk patients. Secondly, the observed associations with a younger age, female gender, and a history of blood transfusion highlight possible future target groups for screening and follow-up. Thirdly, despite the overall low prevalence and mild disease burden observed, the potential for aggressive HDV-related liver disease remains, requiring routine anti-HDV testing in certain situations, particularly before initiating HBV antiviral therapy or performing liver transplantation.

In conclusion, this study provides the first contemporary, nationally representative estimate of HDV exposure among Omani HBsAg-positive patients, reporting a prevalence of 5.63%. While prior exposure was evident, active HDV replication was rare, and most anti-HDV–positive patients had only mild fibrosis. The history of blood transfusion is a key predictor of anti-HDV positivity. Despite the overall low burden of active HDV infection, the potential for progressive disease underscores the need for continued surveillance. A targeted screening approach, particularly in high-risk groups, may represent the most cost-effective strategy for Oman, while routine testing before HBV antiviral therapy or transplantation remains essential.

## Figures and Tables

**Table 1:** Comparison between subgroups of the studied population according to their anti-HDV status.

Variables		Total Cohort n = 639 (%)	Negative for HDV n = 603 (%)	Positive for HDV n = 36 (%)	*p*-Value **
Demographic	Age (mean ± SD)	46.61 ± 8.76 Range of 28–85	46.77 ± 8.80	44.00 ± 7.73	0.07
	Male gender	379 (59.31)	372 (61.69)	7(19.44)	<0.001
	Married	623 (97.50)	587 (97.35)	36 (100.00)	0.32
Clinical	DM	47 (7.36)	45 (7.46)	2 (5.56)	0.67
	Hypertension	35 (5.48)	33 (5.47)	2 (5.56)	0.98
	Dyslipidemia	18 (2.82)	18 (2.99)	0 (0.00)	0.29
	CKD	3 (0.47)	3 (0.50)	0 (0.00)	0.67
	Liver transplantation	7 (1.10)	6 (1.00)	1 (2.78)	0.32
	MASLD	266 (41.69)	253 (41.96)	13 (37.14)	0.57
	HCC	3 (0.47)	3 (0.50)	0 (0.00)	0.67
	HBV treatment	109 (17.06)	106 (17.58)	3 (8.33)	0.15
Risk factors	Travel to the endemic area	373 (58.37)	355 (58.87)	18 (50.00)	0.29
	Family history of HBV	93 (14.55)	86 (14.26)	7 (19.44)	0.39
	Blood transfusion	6 (0.94)	3 (0.50)	3 (8.33)	<0.001
	History of surgery	64 (10.02)	57 (9.45)	7 (19.44)	0.05
	Alcohol intake	7 (1.10)	7 (1.16)	0 (0.00)	0.52
	Smoking	4 (0.63)	4 (0.66)	0 (0.00)	0.62
Co-infection	HCV	5 (0.78)	5 (0.83)	0 (0.00)	0.58
	HIV	1 (0.16)	1 (0.17)	0 (0.00)	0.81
Investigations (mean ± SD)	Total bilirubin (µmol/L)	9.12 ± 8.77	9.19 ± 8.93	7.91 ± 5.41	0.39
	Serum albumin (g/L)	43 ± 5	43 ± 5	42 ± 4	0.16
	ALT (IU/L)	30 ±28	30 ± 29	24 ± 16	0.15
	AST (IU/L)	26 ± 17	26 ± 17	24± 19	0.69
	ALP (IU/L)	73 ± 28	74 ± 28	68 ± 25	0.28
	HBe Ag	32 (5.01)	31 (5.14)	1 (2.78)	0.53
	HBVDNA (log)	3.18 ± 1.42	3.19 ± 1.43	3.03 ± 1.42	0.55
Fibrosis *^,†^	F0–F1	596 (93.27)	560 (92.87)	36 (100)	0.43
	F2	27 (4.23)	27 (4.48)	0 (0.00)	
	F3	3 (0.47)	3 (0.50)	0 (0.00)	
	F4	13 (2.03)	13 (2.16)	0 (0.00)	

Abbreviations: HDV, hepatitis D virus; DM, Diabetes Mellitus; CKD, Chronic Kidney Disease; MASLD, Metabolic Dysfunction-Associated Steatotic Liver Disease; HCC, hepatocellular carcinoma; HBV, hepatitis B virus; HCV, hepatitis C virus; HIV, Human Immunodeficiency Virus; ALT, Alanine Aminotransferase; AST, Aspartate Aminotransferase; ALP, Alkaline Phosphatase; F0–F4, fibrosis stages. SD, standard deviation. * Fibrosis stage is based on liver stiffness measurement (LSM) with FibroScan^®^ device using vibration-controlled transient elastography (VCTE). ** *p*-value of differences between HDV-positive and -negative patients. ^†^ LSM is not validated in HBV-related liver transplant patients.

**Table 2:** Univariate logistic regression analysis of factors associated with anti-HDV positivity.

Variable	Univariate Regression			Ridge Regression
	OR	95% CI	*p*-Value	OR
Age	0.96	0.92–1.00	0.07	0.96
Male gender	0.15	0.06–0.35	<0.001	0.15
Blood transfusion (Yes)	18.18	3.53–93.56	0.001	19.94
Travel to the endemic area (Yes)	0.70	0.36–1.37	0.30	0.63

## Data Availability

The raw data supporting the conclusions of this article will be made available by authors on request.
